# Supplementary material for: Characterization of Unidirectional Replication Forks in the Mouse Genome
Source: Int J Mol Sci. 2023 Jun 1;24(11):9611. doi: 10.3390/ijms24119611 (PMC10253849; doi:10.3390/ijms24119611)
Supplement: Supplementary file 1 [file ijms-24-09611-s001.zip › ijms-2401437-supplementary.pdf]

Supplementary Materials

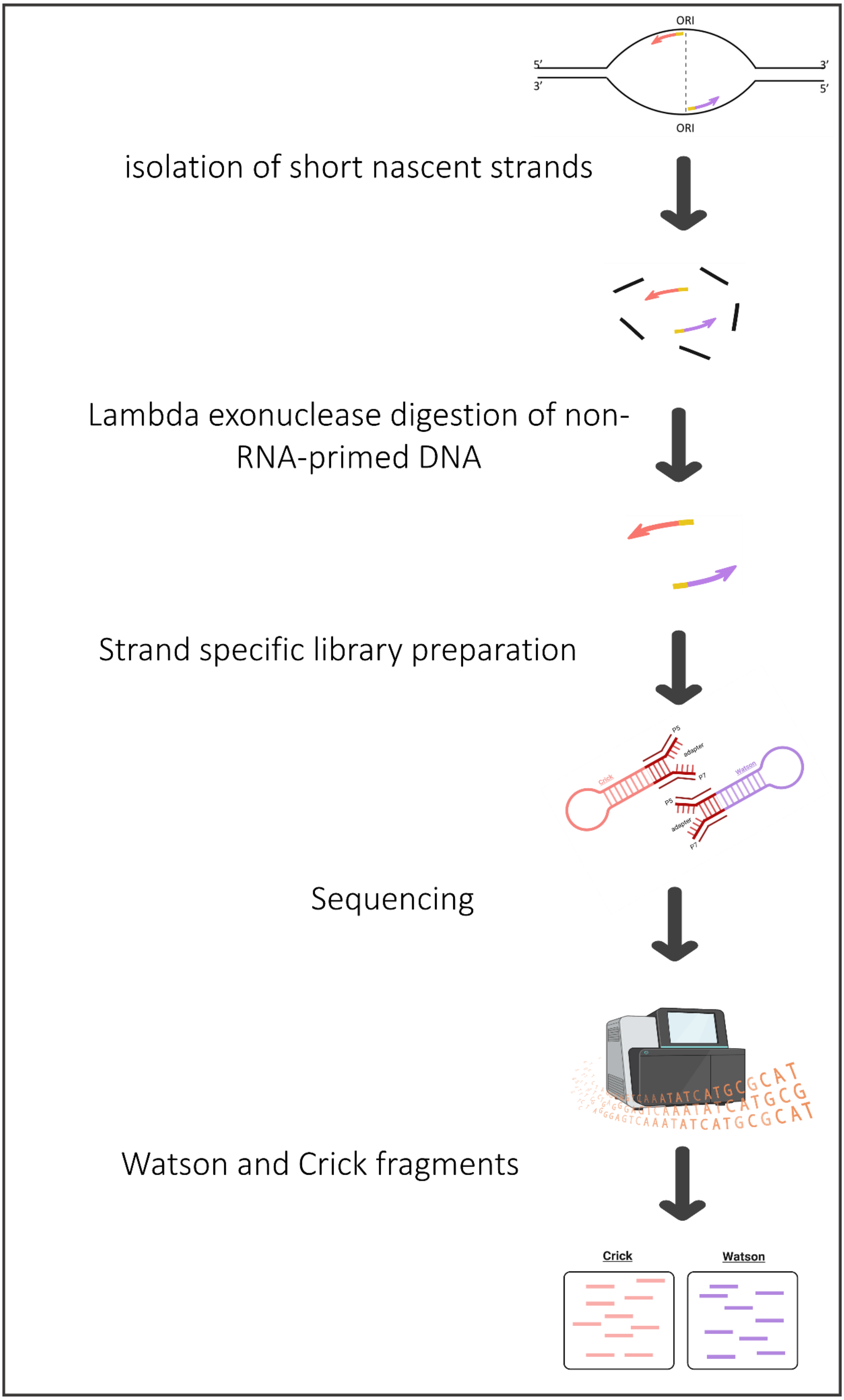

Figure S1. Schematic of the Ori-SSDS protocol to sequence nascent leading strands.

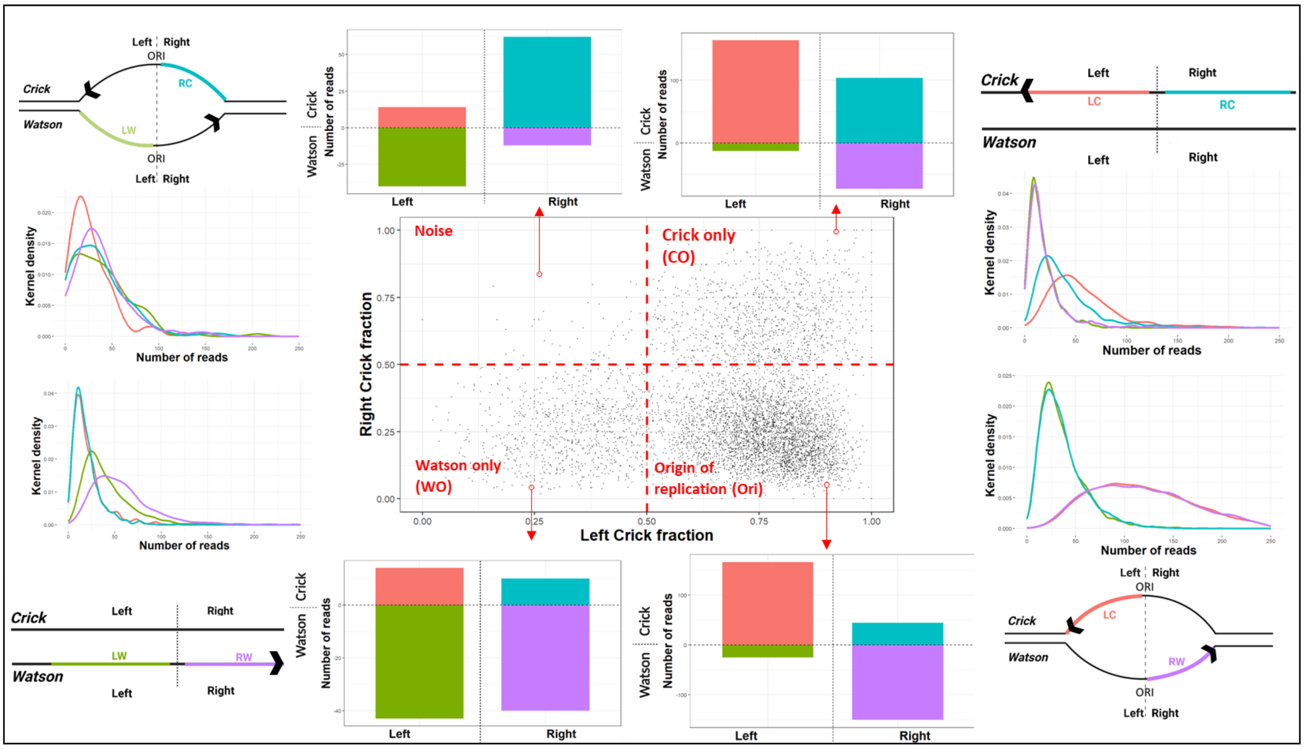

Figure S2. **Using Ori-SSDS data for the identification of non-symmetric origins in testis.** Same as figure 1B for testis Ori-SSDS data.

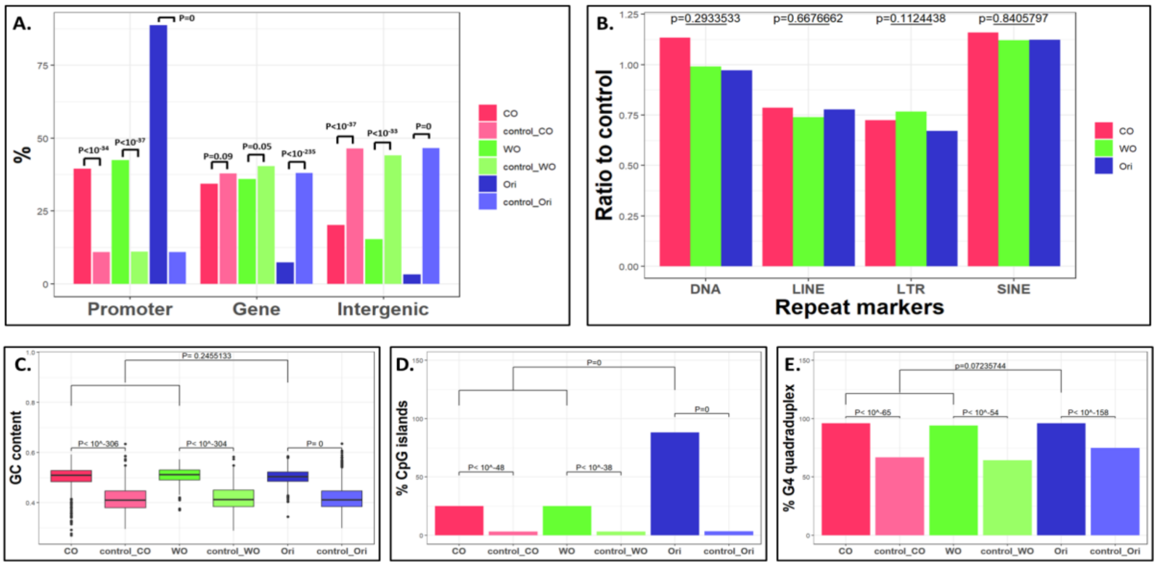

Figure S3. Genetic characterization of the three types of peaks in testis.

Same as figure 2 for testis data.

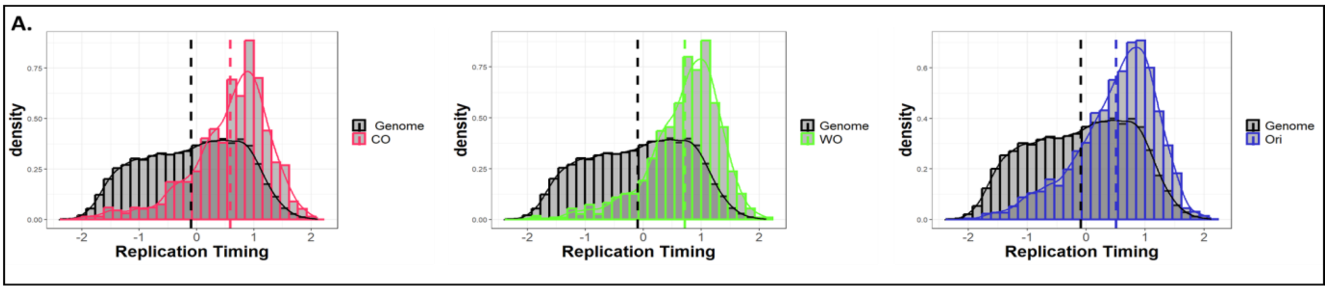

Figure S4. Replication timing characterization of the three types of peaks in testis. Sama as Figure 3C for testis. All three distributions were extremely different than the distribution of the RT in the entire genome (P~0; KS test). The distribution of RT of the origins was significantly different than that of WO (P<0.004; KS test), but not of CO.

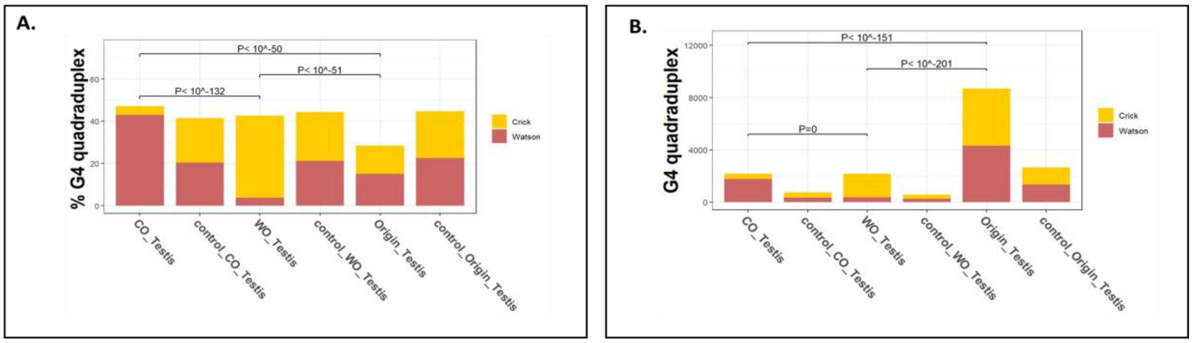

Figure S5. G4 quadruplexes distinguishes between the unidirectional and bidirectional origins in testis. Same as figure 4a-b for testis.

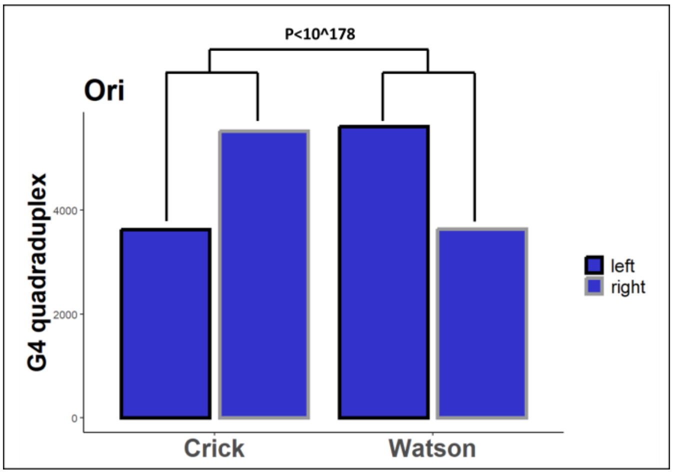

Figure S6. G4 quadruplexes are enriched for lagging strand synthesis. The amount of G4 quadruplexes that were mapped to the Crick and Watson strands 2 Kb 5' and 3' to the center of the Ori peaks in ES cells. The statistical significance of the differences was assessed using the Chi-square test.

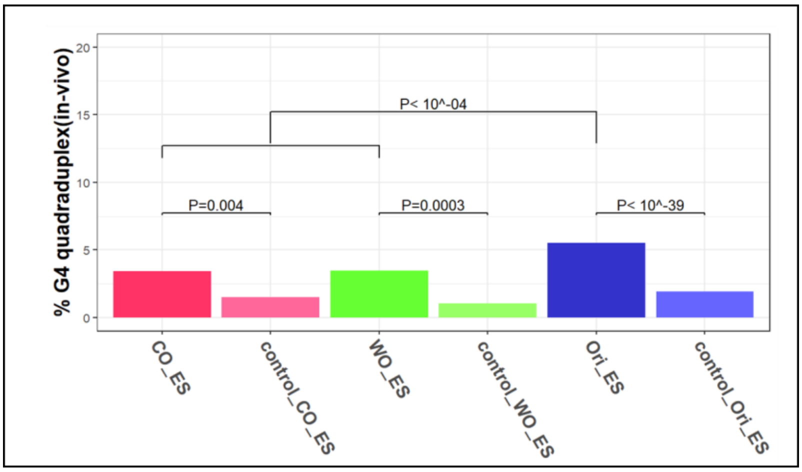

Figure S7. In vivo G4 quadruplex are enriched around the three types of peaks in ES cells. Bar graph showing the percentage of each type of peaks and its corresponding control that overlaps with G4 quadruplex structures, using cut and tag data. The statistical significance of the differences was assessed using the Chi-square test.

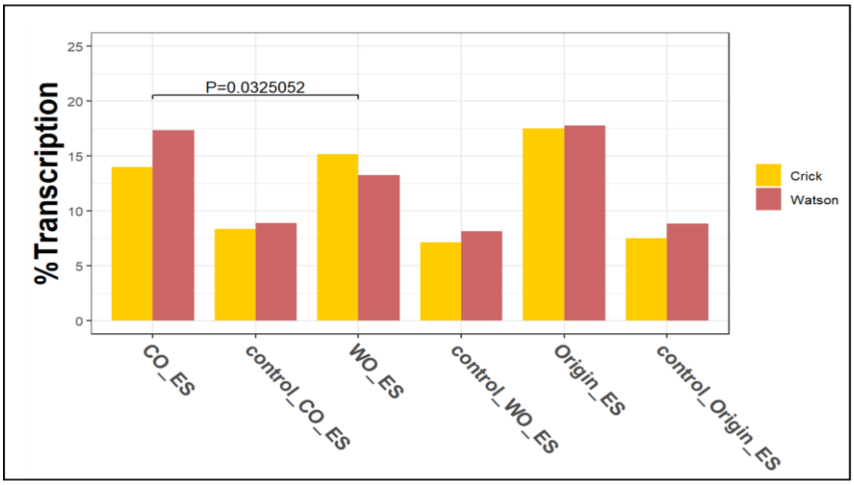

Figure S8. transcription is preferably associated with replication in the head to tail configuration in ES cells. Bar graph representations of the percentage of peaks that overlap with transcribed genes (RPKM>5) separated by the transcribed strand. The statistical significance was assessed by Chi-square test. The designated strand is the coding strand thus Watson transcription is actually carried on the Crick strand (as the template strand) and thus it is Head to head with the leading strand replication on the Crick strand.

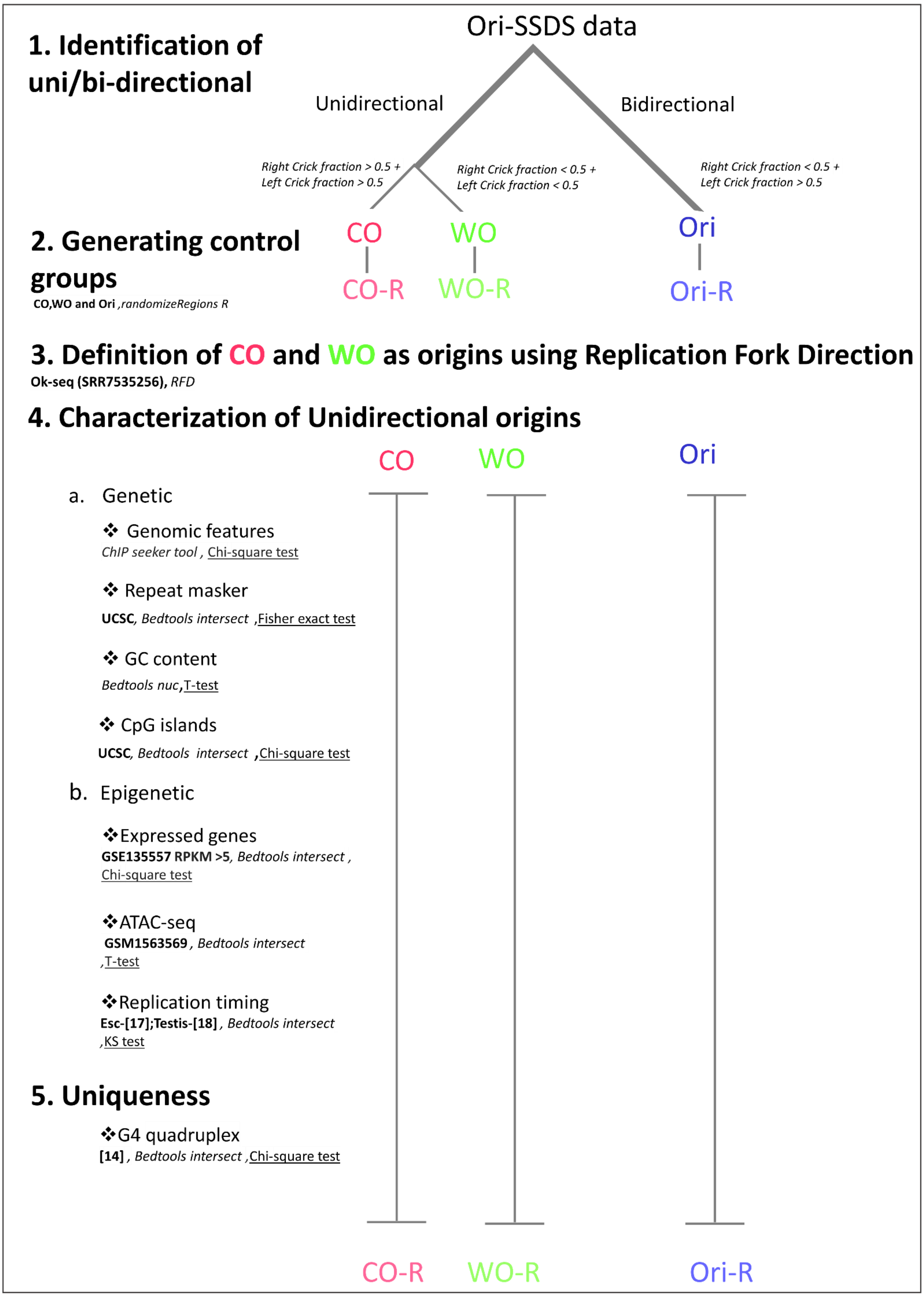

Figure S9. Schematic workflow of the computational analysis

A schematic representation of all the analyses performed on the data. For each analysis step, the dataset used is written in **bold;** the analysis tool or methodology is written in *Italics*; and in the statistical test is underlined.

CO represents Crick only unidirectional peaks,WO represents Watson only unidirectional peaks and Ori represents bidirectional origins of replication.

CO-R, WO-R and Ori-R represent control_CO, control_WO and control_Ori, respectively.
